# Vaccination reduces viral load and accelerates viral clearance in SARS-CoV-2 Delta variant-infected patients

**DOI:** 10.1080/07853890.2023.2166681

**Published:** 2023-03-02

**Authors:** Hongxia Li, Yanzi Li, Junhui Liu, Jianlin Liu, Jianfeng Han, Lin Yang

**Affiliations:** a Department of Medical Administration, First Affiliated Hospital of Xi’an Jiaotong University, Xi’an, China; b Department of Clinical Laboratory, First Affiliated Hospital of Xi’an Jiaotong University, Xi’an, China; c Department of Vascular Surgery, First Affiliated Hospital of Xi’an Jiaotong University, Xi’an, China; d Department of Administrative Office, First Affiliated Hospital of Xi’an Jiaotong University, Xi’an, China

**Keywords:** SARS-CoV-2, delta variant, vaccine, protection effect, outcomes

## Abstract

**Objective:**

The purpose of this study was to investigate vaccine effectiveness in relieving symptoms in patients with the SARS-CoV-2 delta (B.1.617.2) variant.

**Methods:**

In this retrospective study, 31 patients did not receive any vaccine (non-vaccination, NV), 21 patients received 1-dose of inactivated vaccine (one-dose vaccination, OV), and 60 patients received at least 2-dose inactivated vaccine (two-dose vaccination, TV). The baseline data, clinical outcomes and vaccination information were collected and analyzed.

**Results:**

Patients in the OV group were younger than those in the other two groups (*p* = 0.001), but there was no significant difference in any of the other baseline data among the three groups. The TV group showed higher IgG antibody levels and cycle threshold values of SARS-CoV-2 than the NV and OV groups (*p* < 0.01), and time to peak viral load was shorter in the TV group (3.5 ± 2.3 d) than in the NV (4.8 ± 2.8 d) and OV groups (4.8 ± 2.9 d, *p* = 0.03). The patients in the TV group (18%) showed a higher recovery rate without drug therapy (*p* < 0.001). Viral clearance time and hospital stay were significantly shorter in the TV group than in the NV and OV groups (*p* < 0.01), and there were no significant differences in these parameters between the OV and NV groups, but IgG values were higher in the OV group (*p* = 0.025). No severe complications occurred in this study.

**Conclusions:**

Our results suggest that 2-dose vaccination can reduce viral load and accelerate viral clearance in patients with the delta variant and enhance the protection afforded by IgG antibodies *in vivo*.

**Key Messages**
- In this study, our results shows that two-dose vaccination can reduce viral loads and accelerate viral clearance, and two-dose vaccination enhance the protection of IgG antibodies in vivo; however, one-dose vaccination did not confer protective effectiveness.

## Introduction

Coronavirus disease 2019 (COVID-19) is oneof the most serious global public health crisis in this century, and severe acute respiratory syndrome coronavirus 2 (SARS-CoV-2) outbreaks have caused millions of deaths and placed heavy burdens on individuals and societies [1]. Therefore, vaccination is an accepted strategy for protecting individuals against severe COVID-19 and its consequences and is an important key step in controlling the pandemic of SARS-CoV-2 infection. However, with the emergence of various variants of the SARS-CoV-2 virus, the delta variant quickly became the main viral strain of the pandemic [2–5]. In addition, there is some debate about the protective effect of vaccines against the delta variant. Some studies have confirmed vaccine effectiveness in strictly selected populations, and these data suggested that the different vaccines could protect against the delta variant and reduce viral infection [6–9]. More importantly, in the course of the SARS-cov-2 pandemic, we need to make exact diagnosis based on the multiple nucleic acid test results and medical history combined with radiological examination and laboratory examination of the patients, rather than the single nucleic acid test results, which helps to avoid misdiagnosis and missed diagnosis of COVID-19 case. Accurate diagnosis is conducive to more accurate epidemiological management and more authentic evaluation of the effect of vaccines [10–13].

Previous reports have shown that vaccination is effective in reducing household transmission of the alpha variant by 40–50%, with viral load in the upper respiratory tract significantly lower in vaccinated patients [14,15]. Other research has revealed that vaccines could have a moderate to a high protective effect against the delta variant [16,17]. Previous reports suggested that more than half of people may be at risk of a likely severe COVID19, because of their underlying medical issues and risk factors [18], therefore, it is urgent to strengthen the vaccination of the whole population, especially those with basic diseases. Due to the limitations of the general population’s access to information on vaccines, some studies have proved that slightly more than half of the population received vaccination due to work, social life et al. [19].

Evaluating the vaccines against SARS cov-2 variants is the focus of the current pandemic, and it is equally important to take preventive measures to prevent infection [20]. In the face of mutating viruses, it is important to evaluate the protective effect of existing vaccines in the real world, which is helpful to quickly adjust public policies and develop new vaccines. However, there are few data on the actual protective effect of the vaccine in patients infected with the delta variant in the real world. Therefore, in this study, we performed a real-world study of the protective effect of the inactivated vaccine in patients infected with the delta variant in northwestern China.

## Methods

### Patients and study design

**Figure 1.**
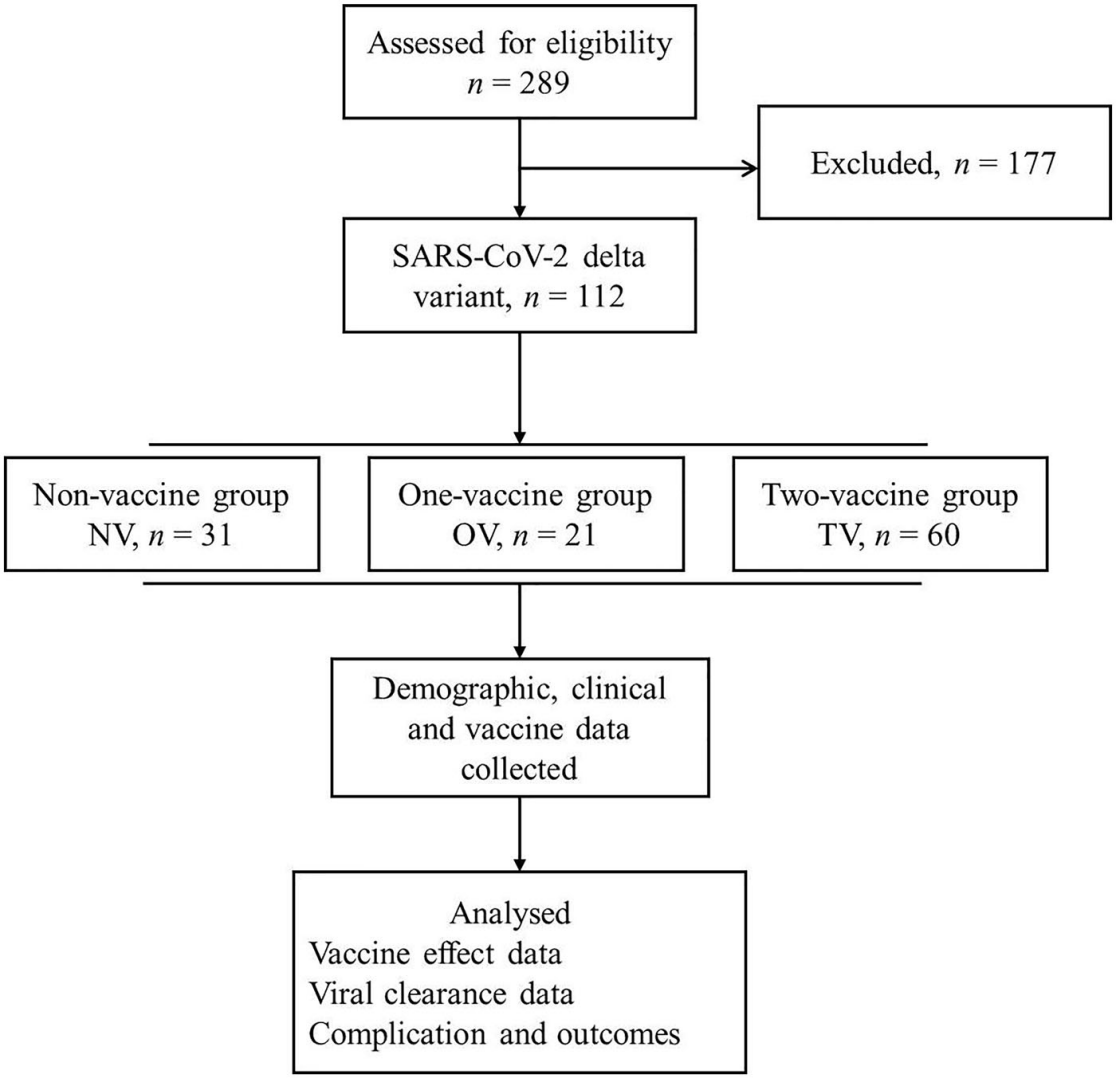
The flowchart of the study. NV, non-vaccination group; OV, one-dose vaccination; TV, two-dose vaccination.

All patients were treated by the medical team of the First Affiliated Hospital of Xi’an Jiaotong University between 20 December 2021, and 20 January 2022. The eligible discharged patients were screened in this retrospective study (Figure 1), and patients younger than 5 years old were excluded. Patients with incomplete and inaccurate baseline data were also excluded from this study. All patients were confirmed to infect with SARS-CoV-2 delta variant by Xi’an CDC (Center for Disease Control and Prevention). There were 31 patients who did not receive any vaccination (non-vaccination group, NV), 21 patients who received only one-dose vaccination (one-vaccination group, OV) and 60 patients who received two- or three-dose vaccination (two-vaccination group, TV). All vaccinated patients received inactivated vaccines (inactivated SARS-CoV-2 vaccine CoronaVac, and aluminium hydroxide as adjuvant. Sinovac life sciences Co. Ltd.). All patients underwent nasopharyngeal swab sample collection according to the national guidelines [16] and were diagnosed *via* SARS-CoV-2 nucleic acid amplification tests, and the results were confirmed by the Xi’an Center for Disease Control and Prevention. This study was approved by the Ethics Committee of the First Affiliated Hospital of Xi’an Jiaotong University, and all patients provided written informed consent. All research methods were carried out in compliance with the relevant declarations of medical ethics and the Declaration of Helsinki.

### Data collection

The collected data included demographic characteristics, epidemiological data (direct and indirect contact history, incubation period), clinical data, chest computed tomography (CT), SARS-CoV-2 detection information, antiviral antibody information, complications and outcomes. All data were obtained from the electronic medical record system, and no follow-up data were included in this study. Vaccination information was collected from the patients, including vaccine name, dose, and date of administration. Two-dose vaccination was defined as having received a second or third dose of vaccine at least 14 days prior; one-dose vaccination was defined as having received the first dose of the vaccine at least 21 days ago but not the second dose. Unvaccinated patients did not receive any SARS-CoV-2 vaccine. Antibodies against SARS-CoV-2 (anti-Spike IgG and IgM) were detected by laboratory tests. All data were extracted by two independent physicians, and any disputed data were resolved in consultation with a third independent physician.

### SARS-CoV-2 nucleic acid and antibody tests

SARS-CoV-2 nucleic acid expression was detected with SARS-CoV-2 virus kits *via* quantitative reverse transcription polymerase chain reaction (2019-nCoV Nucleic acid detection Kit, Daan Gene, Guangzhou, China) according to the national guideline and the manufacturer’s instructions, and all patients were examined daily during hospitalization. The conditions for SARS-CoV-2 nucleic acid amplification were 50 °C for 15 min, 95 °C for 15 min, followed by 45 cycles of 94 °C for 15 s and 55 °C for 45 s. Positive detection was defined as a cycle threshold (Ct value) less than 40(500 copies/ml), and the test procedure was carried out in strict accordance with the protocol. A patient’s viral load was defined as the patient’s lowest Ct value during hospitalization, and the time to peak viral load was defined as the time from the first positive SARS-CoV-2 test to the lowest Ct value during hospitalization. The incubation period was defined as the number of days from contact exposure to the onset of a positive nucleic acid result.

Serum antibodies (anti-Spike IgG and IgM) against SARS-CoV-2 were detected *via* a commercial ELISA kit (SARS-CoV-2 Ig G and SARS-CoV-2 IgM, Maccura Biotechnology Co., Ltd., Chengdu, China) according to the manufacturer’s instructions, and the antibodies were detected at admission, day 7, day 10, day 14 and at discharge. Briefly, 96-well plates were coated with purified SARS-CoV-2 antigen in phosphate buffer overnight at 4 °C in physiological saline (PBS). We added 10 µl of each serum sample to a reaction plate, mixed it with 50 µl magnetic beads and 50 µl buffer, incubated it in the reaction plate for 10 min and then washed it. Then, we added 100 µl acridine ester-labeled recombinant magnetic beads and incubated them in the reaction plate for 10 min. After washing, we added the substrate solution and mixed it well to detect the luminescence signal value. The assay detects antibodies to the spike protein of SARS-CoV-2 with a cut-off (CO) value of <1.0 for negative results and the value ≥1.0 were defined as positive, the final value of the test was calculated with the optical density value of the sample/CO value.

### Clinical management and outcome

All patients were treated in an isolation hospital according to the national guidelines. Briefly, therapeutic strategies were selected according to the severity of the symptoms and comorbidities of the patients. Patients with mild symptoms and no pulmonary imaging changes were given prone position ventilation and drug therapy for underlying diseases, and patients with obvious symptoms (with pulmonary imaging changes) were selectively given thymalfasin (1.6 mg, one dose) and lopinavir/ritonavir (250 mg twice daily). Neutralizing antibodies or convalescent plasma were selectively used for severe patients.

SARS-CoV-2 patients were classified into four types: mild, common, severe and critical, the clinical classification of SARS-CoV-2 was defined previously by the National Health Commission of China [21,22]. A patient whose symptoms disappeared during hospitalization and who was negative by two consecutive nasopharyngeal nucleic acid tests (separated by an interval of at least 24 h) could be discharged from the hospital. The time from a patient’s first positive SARS-CoV-2 test to the end of the two consecutive negative tests was defined as the viral clearance time. Time to peak viral load was defined as the time from the first positive test to the lowest Ct value during hospitalization. The main adverse events were defined as death from any cause, myocardial infarction, stroke, and deep vein thrombosis that occurred during hospitalization.

### Statistical analysis

All data were collected with Excel file (Version 2013, Microsoft, Redmond, Washington) and analyzed by using SPSS v. 22.0 (SPSS, Chicago, IL, USA) software. Quantitative variables were presented as mean and standard deviation and were tested for normality first, and then hypothesis testing was performed with paired *t* tests and analysis of variance (ANOVA). Categorical variables were presented as frequency and percentages and were analyzed using chi-square tests or the Kruskal–Wallis test, Mann–Whitney *U* test or Fisher exact test. *p* values <0.05 were considered statistically significant.

## Results

### The baseline characteristics of patients in the three groups

There were 112 patients with the SARS-CoV-2 delta variant included in this retrospective cross-sectional study (Figure 1), and their baseline characteristic data are listed in Table 1. Our results showed that the patients in the OV group were younger than those in the other two groups (*p* = 0.001), and no significant differences were confirmed for the other baseline characteristics. Except for the incidence of intestinal symptoms, which was higher in the NV group than in the other two groups (*p* = 0.02), there were no significant differences in the other symptoms and comorbidities, and the most common symptoms included cough, sputum production, sore throat, anosmia and dysgeusia (*p* > 0.05); however, the incidence of any symptoms in TV group was significantly lower than NV and OV groups (*p* < 0.05).

**Table 1.** Baseline characteristics of patients in three groups.

	NV group (*n* = 31)	OV group (*n* = 21)	TV group (*n* = 60)	*p* value
Gender (M)	13 (42)	15 (71)	37 (62)	0.08
Age (years)	36.9 ± 13.8	22.5 ± 13.9	33.7 ± 13.7	0.001
BMI (Kg/ m^2^)	22.7 ± 3.6	22.0 ± 5.8	23.2 ± 3.6	0.53
Comorbidities				0.32
Hypertension	2 (7)	0 (0)	1 (2)	
Cirrhosis	2 (7)	0 (0)	1 (2)	
Operation history	1 (3)	1 (5)	1 (2)	
COPD	1 (3)	0 (0)	1 (2)	
Type 2 diabetes	0 (0)	2 (10)	1 (2)	
Any symptoms	31 (100)	16 (76)	33 (55)	<0.05
Stuffy nose/snot	1 (3)	1 (5)	1 (2)	0.74
Sore throat	4 (13)	1 (5)	3 (5)	0.35
Cough	14 (45)	7 (33)	20 (33)	0.52
Sputum production	8 (26)	4 (19)	5 (8)	0.08
Dyspnea	1 (3)	0 (0)	1 (2)	0.69
Intestinal symptom	3 (10)	0 (0)	0 (0)	0.02
Anosmia/dysgeusia	3 (10)	3 (14)	2 (3)	0.20
Fatigue	0 (0)	0 (0)	1 (2)	0.65

Data are presented as n (%) or mean ± standard deviation. NV: non-vaccines; OV: one-vacine; TV: two-vaccine; M: male; kg: kilogram; BMI: body mass index; COPD: chronic obstructive pulmonary disease. *p* value, comparison among groups.

### Clinical results in the three groups

**Figure 2.**
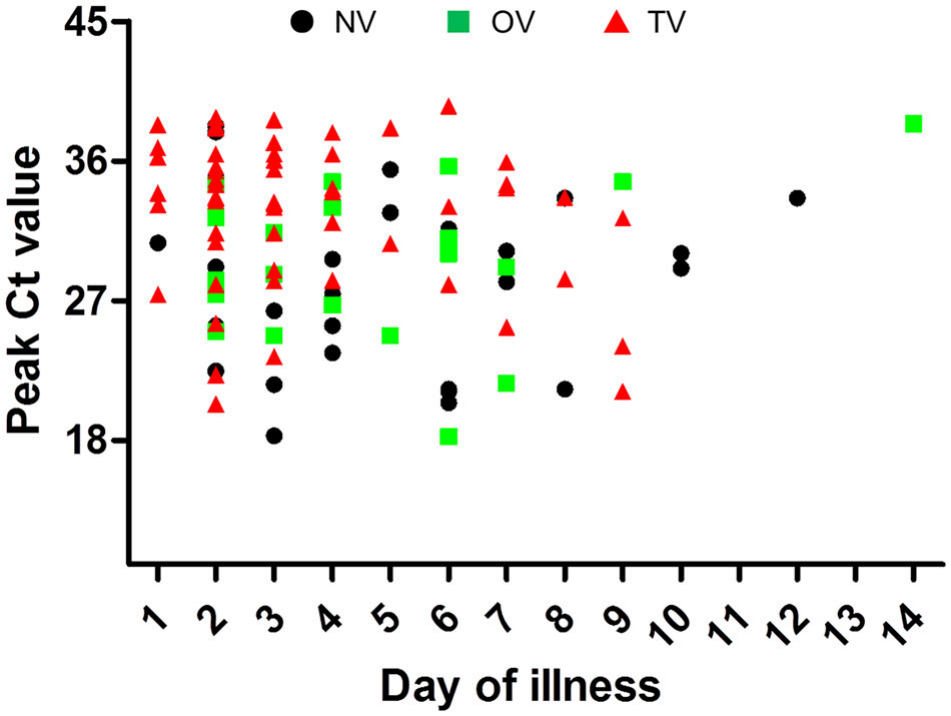
Scatterplot of peak Ct values and time to peak of viral load among three groups. NV: non-vaccination group (black circle); OV: one-dose vaccination (green square); TV: two-dose vaccination (red triangle), the TV group showed the lower viral load and shorter time to peak than the NV and OV groups (*p* < 0.05).

All clinical and epidemiological data are listed in Table 2 and Table 3. All patients had mild or common SARS-CoV-2 infections, and no severe or critical cases occurred in the three groups, and the patients showed a similar contact history. The chest CT and C-reactive protein and IgM levels were similar among the three groups (*p* > 0.05). The TV group showed a similar incubation period of SARS-CoV-2 as the NV and OV groups. However, the cycle threshold value of SARS-CoV-2 was higher in the TV group (32.4 ± 5.1) than in the NV (28.4 ± 5.7) and OV groups (29.4 ± 5.4, *p* < 0.01), and time to peak viral load were shorter in the TV group (3.5 ± 2.3 d) than in the NV (4.8 ± 2.8 d) and OV groups (4.8 ± 2.9 d, *p* = 0.03, Figure 2). Moreover, levels of the antibody IgG were also higher in the TV group (7.2 ± 3.3) than in the NV (2.4 ± 3.2) and OV groups (4.7 ± 3.9, *p* < 0.001) and higher in the OV group than in the NV group (*p* = 0.025). Most patients underwent the therapeutic strategies described in the Methods section, and the patients in the TV group (18%) showed a higher recovery rate without drug therapy (13% of NV, 10% of OV, *p* < 0.001; Table 3). There were no significant differences in the clinical parameters between the NV and OV groups (*p* > 0.05).

**Table 2.** The clinical results of patients in three groups.

	NV group (*n* = 31)	OV group (*n* = 21)	TV group (*n* = 60)	*p* value
Contact history				0.43
Direct contact	28 (90)	19 (91)	49 (82)	
Indirect contact	3 (10)	2 (10)	11 (18)	
Clustered feature	25 (81)	16 (76)	42 (70)	0.54
CT positive	21 (68)	14 (67)	30 (50)	0.25
Classification				0.91
Mild	9 (29)	7 (33)	20 (33)	
Common	22 (71)	14 (67)	40 (67)	
Severe/critical	0 (0)	0 (0)	0 (0)	
Cycle threshold	28.4 ± 5.7	29.4 ± 5.4	32.4 ± 5.1	<0.01
Time to peak (d)	4.8 ± 2.8	4.8 ± 2.9	3.5 ± 2.3	0.03
Antibody value				
IgG (S/CO)	2.4 ± 3.2	4.7 ± 3.9	7.2 ± 3.3	<0.001
Ig M (S/CO)	3.2 ± 9.6	4.1 ± 13.9	7.6 ± 18.1	0.39
CRP positive	8 (26)	7 (33)	17 (28)	0.84

Data are presented as *n* (%) or mean ± standard deviation. NV: non-vaccines; OV: one-vacine; TV: two-vaccine; CRP: C reactive protein. *p* value, comparison among groups.

**Table 3.** The outcomes of patients in three groups.

	NV group (*n* = 31)	OV group (*n* = 21)	TV group (*n* = 60)	*p* value
Oxygen support	27 (87)	19 (91)	58 (97)	0.22
No-drug used	4 (13)	2 (10)	11 (18)	<0.001
Anticoagulation	16 (52)	11 (52)	36 (60)	0.69
Antibodies/convalescent plasma	0 (0)	0 (0)	0 (0)	NA
Thymalfasin	2 (7)	2 (10)	4 (7)	0.89
Lopinavir/ritonavir	0 (0)	0 (0)	1 (2)	0.65
Hospital stay (d)	17.6 ± 4.4	17.1 ± 3.5	14.4 ± 2.2	<0.01
Viral clearance time (d)	14.3 ± 3.6	13.8 ± 3.6	11.2 ± 1.9	<0.001
Time to peak (d)	4.8 ± 2.8	4.8 ± 2.9	3.5 ± 2.2	0.03
Symptom recovery				
Stuffy nose/snot	0 (0)	0 (0)	0 (0)	NA
Sore throat	0 (0)	0 (0)	0 (0)	NA
Cough	0 (0)	0 (0)	0 (0)	NA
Sputum production	0 (0)	0 (0)	0 (0)	NA
Dyspnea	0 (0)	0 (0)	0 (0)	NA
Intestinal symptom	0 (0)	0 (0)	0 (0)	NA
Anosmia/dysgeusia	1 (3)	1 (5)	1 (2)	0.74
Fatigue	0 (0)	0 (0)	0 (0)	NA
Death/ARDS	0 (0)	0 (0)	0 (0)	NA
MI/Stroke	0 (0)	0 (0)	0 (0)	NA
DVT	0 (0)	0 (0)	0 (0)	NA

Data are presented as n (%) or mean ± standard deviation. NV: non-vaccines; OV: one-vacine; TV: two-vaccine; ARDS: acute respiratory distress syndrome; MI: myocardial infarction; DVT: deep vein thrombosis. *p* value, comparison among groups.

### Patient outcomes in the three groups

The outcome parameters after isolation therapy are shown in Table 3. One patient in each of the three groups still had mild anosmia/dysgeusia, but their remaining symptoms disappeared completely (*p* > 0.05). During therapy, SARS-CoV-2 viral clearance time was significantly shorter in the TV group (11.2 ± 1.9 d) than in the NV (14.3 ± 3.6 d) and OV groups (13.8 ± 3.6 d, *p* < 0.001). Moreover, the hospital stays of patients in the TV group (14.4 ± 2.2 d) were shorter than those of patients in the NV (17.6 ± 4.4 d) and OV groups (17.1 ± 3.5 d, *p* < 0.01). There were no significant differences in these parameters between the NV and OV groups (*p* > 0.05). No severe adverse events (death, stroke, acute respiratory distress syndrome, myocardial infarction or deep vein thrombosis) occurred in the three groups.

## Discussion

The spike protein of the delta variant shows a higher binding affinity with the human angiotensin-converting enzyme 2 receptors than that of the spike proteins of earlier variants, suggesting that the delta variant has a high transmission and rapid cell infectivity [23,24]. Thus, patients with the delta variant exhibit significantly higher transmissibility than that of patients infected by earlier variants regardless of vaccination. In our study, we found that incubation periods were shorter in all three groups, which is also consistent with the results of previous studies [25–27]. Vaccination had a significant effect on the occurrence of any symptoms in delta variant patients, especially, the incidence of intestinal symptoms was higher in nonvaccinated patients; meanwhile, the most common symptoms included cough, sore throat and anosmia/dysgeusia. These data suggest that vaccination may help attenuate damage to the gastrointestinal system from the delta variant.

A previous study revealed that vaccination shows a low protective effect against the delta variant except for a satisfactory protective effect against severe infections [28]. There were no significant differences in chest CT findings, systemic inflammation or IgM levels in this study, which seems to indicate that the vaccine provides limited protection against mild and common severity of SARS-CoV-2 delta variant infection in patients. However, we still found an obvious protective effect of the vaccine in patients with the Delta variant. When compared with nonvaccinated or one-dose vaccinated patients, two-dose vaccinated patients showed significantly lower viral load and shorter time to peak viral load, and viral clearance time in these patients was also significantly shortened. Pouwels et al. confirmed that vaccination was able to reduce the incidence of new infections and peak viral burdens [29]. In our study, two-dose vaccinated patients showed a lower incidence of any symptoms, these results confirmed that the in inactivated vaccines still has the certain protective effectiveness on the delta variant, and further research are needed to confirm the difference in the protective effect between the inactivted vaccine and the other vaccines. Although the different vaccines show a protective effect on the various mutants of the virus, existing vaccines are designed for the initial SARS-CoV-2 virus and not its mutants. We need to pay more attention to surveillance the long-term safety and effectiveness of the vaccinated general population, adverse reactions after vaccination should be strictly observed. Some studies reveal that these adverse reactions may be serous [30,31].

Our results confirmed that only two-dose vaccinated patients had lower viral load and higher IgG antibody levels, and the IgG value may be one of the indicators reflecting the protective effect of vaccination. These data indicate that the vaccines remain effective against the delta variant. Previous studies have demonstrated that serum IgG in patients with SARS-CoV-2 infection was closely related to its protective effect, and if the IgG antibody value was less than 50 BAU/mL, the protective effect of the vaccine was very limited [32]. In our study, the IgG antibody levels of the 2-dose vaccinated patients were significantly higher than those of patients in the other two groups, and the viral clearance times were also significantly shortened, indicating that the vaccine still provides significant protection for patients with non-severe delta variant infection. Our results suggest that patients who received two doses of vaccinated may elicit stronger immune responses that relieve the symptoms and damage caused by the delta variant virus infected. Therefore, some patients with mild symptoms were able to recover completely without drug treatment. In this study, we found that the recovery rate without drug treatment in two-dose vaccinated patients reached 18.3%, which was significantly higher than that in the nonvaccine and one-dose vaccine groups. However, the long-term effects and safety of inactivated vaccines still need to be further observed.

Despite vaccine neutralization of the delta variant, the symptom with the slowest recovery is anosmia/dysgeusia; indeed, anosmia/dysgeusia may be a good predictor of SARS-CoV-2 infection [33]. One study showed that partial two-dose vaccinated patients did not develop pneumonia or require additional oxygen support, which suggests that two-dose vaccination can reduce the severity of delta variant infection [34]. Our results are consistent with this report. Moreover, vaccination could accelerate the clearance of the delta variant virus from the respiratory tract, which is beneficial to the recovery of patients [26,35]. However, we need to pay attention to the possibility that some risk factors, such as obesity, diabetes and cancer, may still affect the clinical outcomes of patients [36,37]. In addition, vaccines may differ in protective efficacy, but two-dose vaccination could still significantly reduce the risk of new SARS-CoV-2 infection [38,39]. With the evolution of the SARS-CoV-2 virus, the protective effect of existing vaccines may be reduced, specific vaccine design for different mutant strains may be the focus in the future [31]. With the outbreak of SARS CoV-2 variant strains, the only potential solution is the specific vaccine development targeting against all variant strains to halt its progress. But before that, vaccination remains the first choice to avoid severe infection, and the effect and adverse reaction detection after vaccination also need further attention. While taking preventive control strategies (wearing masks and maintaining social distance) to protect from the virus infection are also equally important [40].

## Limitations

This study has the following limitations. First, due to the vaccination policy, there are increasingly fewer subjects who are not vaccinated; therefore, the numbers of unvaccinated and one-dose vaccinated patients were low in this study, which could cause selection bias and affect the conclusions. Second, this study had a small sample size; we only analyzed the hospitalization data of patients in three specific groups and did not include the data from each patient’s entire disease process, which could also affect the accuracy of the conclusions. Third, we only analyzed the effectiveness of inactivated vaccines and did not include live attenuated vaccines or mRNA vaccines, which may have affected the results. Finally, this study did not analyze the protective effects of vaccines in different populations, which may affect the generalizability of the conclusions.

## Conclusions

In this retrospective study, our results suggest that two-dose vaccination could alleviate symptoms of the SARS-CoV-2 delta variant, reduce viral load and accelerate viral clearance in patients. The two-dose vaccinated patients showed higher IgG antibody levels than the unvaccinated and one-dose vaccinated patients, and the two-dose vaccinated group exhibited a higher recovery rate without the need for medications than the other two groups.

## Data Availability

The datasets used and analyzed during this study are available from the corresponding author upon reasonable request. We confirmed that these patients have not been reported in any other submission by all authors or anyone else.

## References

[1] Gebru AA, Birhanu T, Wendimu E, et al. Global burden of COVID-19: situational analyis and review. Hum Antibodies. 2021;29(2):139–148. 32804122 10.3233/HAB-200420

[2] Supasa P, Zhou D, Dejnirattisai W, et al. Reduced neutralization of SARS-CoV-2 B.1.1.7 variant by convalescent and vaccine sera. Cell. 2021;184(8):2201–2211.e7. 33743891 10.1016/j.cell.2021.02.033 PMC7891044

[3] Thiagarajan K. Why is India having a covid-19 surge? BMJ. 2021;373:n1124. 33931413 10.1136/bmj.n1124

[4] The Lancet India’s COVID-19 emergency. Lancet. 2021;397(10286):1683. 33965073 10.1016/S0140-6736(21)01052-7 PMC8102046

[5] Raman R, Patel KJ, Ranjan K. COVID-19: unmasking emerging SARS-CoV-2 variants, vaccines and therapeutic strategies. Biomolecules. 2021;11(7):993. 34356617 10.3390/biom11070993 PMC8301790

[6] Wall EC, Wu M, Harvey R, et al. Neutralising antibody activity against SARS-CoV-2 VOCs B.1.617.2 and B.1.351 by BNT162b2 vaccination. Lancet. 2021;397(10292):2331–2333. 34090624 10.1016/S0140-6736(21)01290-3 PMC8175044

[7] Wang P, Nair MS, Liu L, et al. Antibody resistance of SARS-CoV-2 variants B.1.351 and B.1.1.7. Nature. 2021;593(7857):130–135. 33684923 10.1038/s41586-021-03398-2

[8] Sheikh A, McMenamin J, Taylor B, et al. SARS-CoV-2 Delta VOC in Scotland: demographics, risk of hospital admission, and vaccine effectiveness. Lancet. 2021;397(10293):2461–2462. 34139198 10.1016/S0140-6736(21)01358-1 PMC8201647

[9] Lopez Bernal J, Andrews N, Gower C, et al. Effectiveness of COVID-19 vaccines against the B.1.617.2 (Delta) variant. N Engl J Med. 2021;385(7):585–594. 34289274 10.1056/NEJMoa2108891 PMC8314739

[10] Mouliou DS, Pantazopoulos I, Gourgoulianis KI. COVID-19 smart diagnosis in the emergency department: all-in in practice. Expert Rev Respir Med. 2022;16(3):263–272. 35245149 10.1080/17476348.2022.2049760 PMC8935450

[11] Mouliou DS, Gourgoulianis KI. COVID-19 ‘asymptomatic’ patients: an old wives’ tale. Expert Rev Respir Med. 2022;16(4):399–407. 35041796 10.1080/17476348.2022.2030224

[12] Mouliou DS, Gourgoulianis KI. False-positive and false-negative COVID-19 cases: respiratory prevention and management strategies, vaccination, and further perspectives. Expert Rev Respir Med. 2021;15(8):993–1002. 33896332 10.1080/17476348.2021.1917389 PMC8074645

[13] Farooqi T, Malik JA, Mulla AH, et al. An overview of SARS-COV-2 epidemiology, mutant variants, vaccines, and management strategies. J Infect Public Health. 2021;14(10):1299–1312. 34429257 10.1016/j.jiph.2021.08.014 PMC8366110

[14] Harris RJ, Hall JA, Zaidi A, et al. Effect of vaccination on household transmission of SARS-CoV-2 in England. N Engl J Med. 2021;385(8):759–760. 34161702 10.1056/NEJMc2107717 PMC8262621

[15] Levine-Tiefenbrun M, Yelin I, Katz R, et al. Initial report of decreased SARS-CoV-2 viral load after inoculation with the BNT162b2 vaccine. Nat Med. 2021;27(5):790–792. 33782619 10.1038/s41591-021-01316-7

[16] Harder T, Külper-Schiek W, Reda S, et al. Effectiveness of COVID-19 vaccines against SARS-CoV-2 infection with the Delta (B.1.617.2) variant: second interim results of a living systematic review and meta-analysis, 1 January to 25 August 2021. Euro Surveill. 2021;26:2100920. 34651577 10.2807/1560-7917.ES.2021.26.41.2100920 PMC8518304

[17] Hughes EC, Amat JAR, Haney J, et al. Severe acute respiratory syndrome coronavirus 2 serosurveillance in a patient population reveals differences in virus exposure and antibody-mediated immunity according to host demography and healthcare setting. J Infect Dis. 2021;223(6):971–980. 33367847 10.1093/infdis/jiaa788 PMC7798933

[18] Mouliou DS, Kotsiou OS, Gourgoulianis KI. Estimates of COVID-19 risk factors among social strata and predictors for a vulnerability to the infection. IJERPH. 2021;18(16):8701. 34444450 10.3390/ijerph18168701 PMC8392732

[19] Mouliou DS, Pantazopoulos I, Gourgoulianis KI. Social response to the vaccine against COVID-19: the underrated power of influence. JPM. 2021;12(1):15. 35055329 10.3390/jpm12010015 PMC8778590

[20] Malik JA, Ahmed S, Mir A, et al. The SARS-CoV-2 mutations versus vaccine effectiveness: new opportunities to new challenges. J Infect Public Health. 2022;15(2):228–240. 35042059 10.1016/j.jiph.2021.12.014 PMC8730674

[21] Zheng C, Chang Z, Liu F, et al. Interpretation of the protocol for prevention and control of COVID-19 in China (Edition 7). China CDC Weekly. 2020;2(47):902–905. 10.46234/ccdcw2020.245 PMC8393151 34594796

[22] NHC. http://www.nhc.gov.cn/yzygj/s7653p/202008/0a7bdf12bd4b46e5bd28ca7f9a7f5e5a/files/a449a3e2e2c94d9a856d5faea2ff0f94.pdf. (accessed Aug 19, 2020).

[23] Zhang J, Xiao T, Cai Y, et al. Membrane fusion and immune evasion by the spike protein of SARS-CoV-2 Delta variant. Science. 2021;374(6573):1353–1360. 34698504 10.1126/science.abl9463 PMC10763652

[24] Kumar V, Singh J, Hasnain SE, et al. Possible link between higher transmissibility of alpha, kappa and delta variants of SARS-CoV-2 and increased structural stability of its spike protein and hACE2 affinity. IJMS. 2021;22(17):9131. 34502041 10.3390/ijms22179131 PMC8431609

[25] Campbell F, Archer B, Laurenson-Schafer H, et al. Increased transmissibility and global spread of SARSCoV-2 variants of concern as at June 2021. Euro Surveill. 2021;26:2100509. 34142653 10.2807/1560-7917.ES.2021.26.24.2100509 PMC8212592

[26] Singanayagam A, Hakki S, Dunning J, et al. ATACCC study investigators. Community transmission and viral load kinetics of the SARS-CoV-2 Delta (B.1.617.2) variant in vaccinated and unvaccinated individuals in the UK: a prospective, longitudinal, cohort study. Lancet Infect Dis. 2021;21:e363. 10.1016/S1473-3099(21)00648-4 PMC8554486 34756186

[27] Grant R, Charmet T, Schaeffer L, et al. Impact of SARS-CoV-2 Delta variant on incubation, transmission settings and vaccine effectiveness: results from a nationwide case-control study in France. Lancet Reg Health Eur. 2022 Feb;13:100278. 10.1016/j.lanepe.2021.100278 PMC8616730 34849500

[28] Cai C, Liu Y, Zeng S, et al. The efficacy of COVID-19 vaccines against the B.1.617.2 (Delta) variant. Mol Ther. 2021;29(10):2890–2892. 34599870 10.1016/j.ymthe.2021.09.024 PMC8486585

[29] Pouwels KB, Pritchard E, Matthews PC, et al. Effect of Delta variant on viral burden and vaccine effectiveness against new SARS-CoV-2 infections in the UK. Nat Med. 2021;27(12):2127–2135. 34650248 10.1038/s41591-021-01548-7 PMC8674129

[30] Mouliou DS, Dardiotis E. Current evidence in SARS-CoV-2 mRNA vaccines and post-vaccination adverse reports: knowns and unknowns. Diagnostics (Basel). 2022;12(7):1555. 10.3390/diagnostics12071555 PMC9316835 35885461

[31] Malik JA, Mulla AH, Farooqi T, et al. Targets and strategies for vaccine development against SARS-CoV-2. Biomed Pharmacother. 2021;137:111254. 33550049 10.1016/j.biopha.2021.111254 PMC7843096

[32] Pierobon A, Zotto AD, Antico A, et al. Outbreak of SARS-CoV-2 B.1.617.2 (Delta) variant in a nursing home 28 weeks after two doses of mRNA anti-Covid-19 vaccines: evidence of a waning immunity. ClinMicrobiol Infect. 2022;28(4):614.e5–614.e7. 10.1016/j.cmi.2021.12.013 PMC8704894 34958917

[33] Trachootham D, Thongyen S, Lam-Ubol A, et al. Simultaneously complete but not partial taste and smell losses were associated with SARS-CoV-2 infection. Int J Infect Dis. 2021;106:329–337. 33819604 10.1016/j.ijid.2021.03.083 PMC8080514

[34] Ong SWX, Chiew CJ, Ang LW, et al. Clinical and virological features of SARS-CoV-2 variants of concern: a retrospctive cohort study comparing B.1.1.7 (Alpha), B.1.315 (Beta), and B.1.617.2 (Delta). Clin Infect Dis. 2022;75(1):e1128–e1136. 34423834 10.1093/cid/ciab721 PMC8522361

[35] Luo CH, Morris CP, Sachithanandham J, et al. Infection with the SARS-CoV-2 Delta variant is associated with higher recovery of infectious virus compared to the Alpha variant in both unvaccinated and vaccinated individuals. Clin Infect Dis. 2022;75(1):e715–e725. 34922338 10.1093/cid/ciab986 PMC8903351

[36] Basse C, Diakite S, Servois V, Institut Curie COVID Group, et al. Characteristics and outcome of SARS-CoV-2 infection in cancer patients. JNCI Cancer Spectr. 2021;5(1):pkaa090. 33604509 10.1093/jncics/pkaa090 PMC7665636

[37] Karimzadeh S, Bhopal R, Nguyen Tien H. Review of infective dose, routes of transmission and outcome of COVID-19 caused by the SARS-COV-2: comparison with other respiratory viruses. Epidemiol Infect. 2021;149:e96. 33849679 10.1017/S0950268821000790 PMC8082124

[38] Souto Ferreira L, Canton O, da Silva RLP, et al. Assessing the best time interval between doses in a two-dose vaccination regimen to reduce the number of deaths in an ongoing epidemic of SARS-CoV-2. PLOS Comput Biol. 2022;18(3):e1009978. 35333872 10.1371/journal.pcbi.1009978 PMC8986122

[39] Embi PJ, Levy ME, Naleway AL, et al. Effectiveness of two-dose vaccination with mRNA COVID-19 vaccines against COVID-19-associated hospitalizations among immunocompromised adults-nine states, January–September 2021. Am J Transplant. 2022;22(1):306–314. 34967121 10.1111/ajt.16641 PMC9805402

[40] Ward MP, Liu Y, Xiao S, et al. Challenges in the control of COVID-19 outbreaks caused by the Delta variant during periods of low humidity: an observational study in Sydney, Australia. Infect Dis Poverty. 2021;10(1):139. 34937575 10.1186/s40249-021-00926-0 PMC8694908

